# Tai Chi exercise improves working memory capacity and emotion regulation ability

**DOI:** 10.3389/fpsyg.2023.1047544

**Published:** 2023-02-17

**Authors:** Yi Wang, Jing Tian, Qingxuan Yang

**Affiliations:** ^1^School of Physical Education, Weinan Normal University, Weinan, China; ^2^School of Foreign Languages, Weinan Normal University, Weinan, China; ^3^Department of Physical Education, Chang'an University, Xi'an, China

**Keywords:** Tai Chi, working memory capacity, emotion regulation, improve, exercise

## Abstract

**Purpose:**

The study aimed to research the promoting effects of Tai Chi exercise on working memory capacity and emotional regulation ability among college students.

**Methods:**

Fifty-five participants were recruited and randomly divided into the Tai Chi group and control group. The Tai Chi group had a 12-week Tai Chi training to implement intervention, while the control group performed non-cognitive traditional sports with the same exercise intensity as the Tai Chi group. The visual 2-back test of action pictures and the Geneva emotional picture system test were performed before and after the trial, which aimed to examine whether the action memory of Tai Chi training can improve individuals’ working memory capacity and emotion regulation ability.

**Results:**

After 12 weeks, a significant difference was observed in Accuracy Rate (AR) (*F* = 54.89, *p* ≤ 0.001) and Response Time (RT) (*F* = 99.45, *p* ≤ 0.001) of individuals’ Visual Memory Capacity between the Tai Chi group and the control group. Significant effects in Time (*F* = 98.62, *p* ≤ 0.001), Group (*F* = 21.43, *p* ≤ 0.001), and Interaction (Groups × time; *F* = 50.81, *p* ≤ 0.001) on Accuracy Rate (AR) of the Visual Memory Capacity were observed. The same effect was observed again on the Response Time (RT) of the Visual Memory Capacity, Time (*F* = 67.21, *p* ≤ 0.001), Group (*F* = 45.68, *p* ≤ 0.001), Interaction (groups × time; *F* = 79.52, *p* ≤ 0.001). Post-hoc analysis showed that at the end of 12 weeks, the participants in the Tai Chi group had significantly higher Visual Memory Capacity than those in the control group (*p* < 0.05).

After 12 weeks, valence difference (*F* = 11.49, *p* ≤ 0.001), arousal difference (*F* = 10.17, *p* ≤ 0.01), and dominance difference (*F* = 13.30, *p* ≤ 0.001) in the emotion response were significantly different between the control group and the Tai Chi group. The effect of valence differences in Time (*F* = 7.28, *p* < 0.01), Group (*F* = 4.16, *p* < 0.05), and Time*Group (*F* = 10.16, *p* < 0.01), respectively, was significant in the Tai Chi group after 12-week intervention. *Post hoc* analysis showed valence swings in the Tai Chi group were significantly lower than that in the control group (*p* < 0.05); The effect of arousal difference in Time (*F* = 5.18, *p* < 0.05), Group (*F* = 7.26, *p* < 0.01), Time*Group (*F* = 4.23, *p* < 0.05), respectively, was significant in the Tai Chi group after 12-week intervention. *Post hoc* analysis showed arousal fluctuations in the Tai Chi group was significantly lower than that in the control group too (*p* < 0.01); As the same, the effect of dominance differences in Time (*F* = 7.92, *p* < 0.01), Group (*F* = 5.82 *p* < 0.05) and Time*Group (*F* = 10.26, *p* < 0.01), respectively was significant in the Tai Chi group. Dominance swings in the Tai Chi group were significantly lower than that in the control group (*p* < 0.001).

**Conclusion:**

The data support our speculation that action memory training in Tai Chi exercise may improve individuals’ working memory capacity, and then improve their emotion regulation ability, which has provided insightful information for customized exercise programs for emotion regulation in adolescents. Thus, we suggest those adolescents who are experiencing volatile moods and poor emotion regulation attend regular Tai Chi classes, which could contribute to their emotional health.

## Introduction

Previous researches found cognitive control is considered to play a central role in emotion regulation (Joormann and Siemer, 2011). There are a growing number of empirical studies also showing that the cognitive control (working memory, attention) is related to the use of various emotion regulation strategies (Pe et al., 2013). Therefore, cognitive impairment represents a core feature of psychiatric disorders such as depression and anxiety disorders, that may be a valuable target of interventions in the future (Kim et al., 2019). Numerous studies have indicated a relationship between working memory (WM) and mental disorders such as anxiety (Kim et al., 2019) and depression (Li et al., 2018; Zhang et al., 2018). Schmeichel et al., 2008 study found that there is a relationship between individual differences in working memory capacity (WMC), and self-regulation in emotional expression and emotional experience (Schmeichel et al., 2008). Individuals with higher WMC are more able to inhibit their negative emotions and express their positive emotions than those with lower WMC. In addition, individuals with high WMC are more likely to evaluate emotional stimuli in a non-emotional way than those with low WMC, so they less respond to emotional stimuli and express their emotions (Schmeichel et al., 2008). Some scholars conclude that this is because patients with mood disorders have a hard time restraining negative information from interfering with them (Baddeley, 2013). Not only do they fail to prevent irrelevant emotional information from entering WM, but they also have difficulty removing task-irrelevant negative information (Foland-Ross et al., 2013). As a result, excessive negative or unpleasant emotions can disrupt WM and decrease attention processing (Ribeiro et al., 2018). Therefore, WM might be a cognitive control centre to process emotion and provide emotional evaluations (Baddeley et al., 2012).

Further Neuroimaging studies have also shown that the neural networks supporting emotion regulation overlap with the front parietal “multi-demand” network implicated in WM performance (Duncan, 2010). Accordingly, there may be a link between these two capacities, that is, improvements in one will lead to improvements in the other by improving general functions of the front parietal network. To prove this hypothesis, participants were asked to have dual *n*-back task training, and after the training, Schweizer compared participants’ pre-training emotion regulation ability and post-training emotion regulation while they were watching movie clips under an MRI scanner. The results of the study showed that at the behavioral level, participants were better able to reduce negative emotional responses after emotional WM training, which is also true at the neural level, that is, individuals who received the training showed greater activation changes in the front parietal network when performing both the emotional WM task and the emotion regulation task. These emotion regulation improvements were associated with greater activity in the targeted front parietal demand network along with other brain regions implicated in affective control, notably the subgenual anterior cingulate cortex (Schweizer et al., 2013). In summary, previous studies have shown that the area of cognition that may be especially susceptible to the effects of emotion is WM, the WM is crucial to modulating the efficacy of emotion regulation (Pe et al., 2013).

Gross’ process model of emotion regulation shows that intervention in emotion regulation can be administered by various ways and forms. However, the most obvious emotion regulation intervention is teaching individuals healthier patterns of emotion regulation. Some interventions that target the general population involve high-level interventions by altering stress awareness (Crum et al., 2013), anxiety awareness (Schweizer et al., 2013), and low-level interventions by improving WM to increase emotion regulation (Schweizer et al., 2013). Low-level interventions *via* enhancing attention and WM are far less complex than high-level interventions, so which is more operable (Engen and Kanske, 2013). Several investigators have therefore focused on emotional regulation ability improvement by training WM, or attention training methods. Emotion regulation can be enhanced through gaze pattern training, according to Wadlinger and Isaacowitz (2011). Renlai Zhou results also suggest that WM training can improve emotion regulation ability (Xiu et al., 2018). Gabrielle study demonstrated that following emotional WM training, WMC could be increased and trait anxiety could be reduced (Veloso and Ty, 2020).

Despite multiple previous attempts, previous studies to improve emotional regulation ability have typically concentrated on WM training and attention training until now. However, these methods have practical limitations in real applications. Therefore, an easier and operable method to improve emotional regulation ability is required.

The benefits of physical exercises are not only limited to physical health, but higher levels of physical activity are also associated with higher levels of cognitive performance (e.g., including memory and attention; Lojovich, 2010). Research has demonstrated that physical exercise has great effect on spatial memory, WM, and executive attention (Cassilhas et al., 2016). Among the different types of physical activity, aerobic exercise below a moderate intensity is particularly noteworthy because it promotes cortical activity, hemodynamics, and metabolism to create a nutritional environment that improves cognitive performance. Therefore, aerobic exercise is effective not only for improving cognitive abilities in adults (Stern et al., 2019), but for cognitive functions improvement in older adults, such as attention, memory, and WM by a meta-analysis (Angevaren et al., 2008; Northey et al., 2018) even when bout physical exercise may be sufficient to improve memory (Roig et al., 2016) and attention (Hogan et al., 2013). Several studies supported this result that physical exercise, especially acute physical exercise, enhances WM for executive functions (Verburgh et al., 2014; Liang et al., 2021), and attention (de Greeff et al., 2018).

In addition to aerobic exercise, there is resistance training which usually includes a variety of different exercises for the upper and lower extremities. Due to its higher variability, the stimulation of the brain is at least similar to that of aerobic exercise. Resistance exercise from moderate to high-intensity can improve cognitive function in older adults (Cheng et al., 2022). Aerobic Exercise for different age groups studies have shown that aerobic exercise is effective for executive function in prepubertal teenagers and older adults (Ludyga et al., 2016). In addition, research has shown that both open and closed skill exercise and other exercise types (e.g., aerobic and resistance exercise) have the same positive effects on cognitive function (Etnier et al., 2020). Therefore, combinations of coordination exercises and cognitive demands (e.g., skill strategies) may result in greater benefits to cognition than either form of exercise alone (e.g., aerobic exercise, coordination exercises, or cognitive demands; Pesce, 2012).

In contrast to one-time acute exercise, long-term chronic exercise has been increasingly studied in recent years with respect to cognitive function. Current research on chronic exercise and cognition focuses mainly on executive function, inhibitory control, and WM. Meaghanh’s study suggests that chronic exercise acts as a moderator of cognitive control and increases the effectiveness of cognitive control in response to various levels of conflict (Wunder and Staines, 2022). Padilla et al. (2013) showed that chronic aerobic exercise was associated with better inhibitory control and an WMC in young adults, suggesting that chronic aerobic exercise promotes cognitive function across the lifespan (Padilla et al., 2014). Etnier reported that the positive effects of long-term exercise on memory function appear to be greater than those of acute exercise. While Roig et al. showed that only long-term exercise improved short-term memory, and similar results were demonstrated in a 2017 review by Rathore and Lom, who showed that long-term training effectively improves short-term and WM performance. This is because chronic exercise participants actively engage in exercise, and active participants mobilize more WM resources and have higher WMC than passive participants (Padilla et al., 2013). Chronic PA can significantly improve WM performance compared to acute exercise (Rathore and Lom, 2017). In conclusion, accumulating evidence suggests that both acute and chronic exercise can improve the performance of various memory systems (Roig et al., 2016).

The current relevant research paradigm comes from three main theories and hypotheses: the executive attention view, the primary-memory/secondary-memory view, and the binding hypothesis. Inhibitory control, a core executive function, is also often used to assess cognitive function. This is because conflict resolution is the most important component of cognitive control (Diamond, 2013). Representative Psychological Tasks which are used to Assess Inhibitory Control, include Stroop task (Mac Leod, 1991), Simon task (Hommel, 2011), and Flanker task (Mullane et al., 2009). The Flanker task is a classic test of inhibitory control, a task that focuses on the influence of conflicting stimulus information and is used to assess executive function. However, because executive function plays a central role in general cognition, it has also been studied to assess cognitive function. For example, the Flanker paradigm was used to assess executive function in elementary school students in a study of a randomized controlled trial of the effects of classroom physical activity on mathematics achievement (Have et al., 2016). In a study evaluating an PA intervention, a modified Erikson flanker task was used to assess cognitive function (Bugge et al., 2014). The Binding Hypothesis of WMC, on the other hand, views WM as a system for building, maintaining, and rapidly updating arbitrary bindings. WM updating tasks should be an excellent metric for WMC because they involve rapid updates of temporary bindings. Participants in tasks such as the *n*-back task must quickly link ordinal or spatial locations and keep them up to date. Updating tasks are closely related to other measures of WMC as well as fluid intelligence, and are therefore particularly well suited to measuring people’s ability to make and maintain temporary links in WM. The ability to store and process information simultaneously is a major limitation of WM. On this basis, complex tasks have been developed to measure WMC (Unsworth et al., 2009). Therefore, WM span tasks-and in particular, counting span, operation span, and reading span tasks are widely used to measures WM capacity (Conway et al., 2005). There is evidence that coactivation of the striatum is triggered during performance of running span and *n*-back tasks (Dahlin et al., 2008), and the striatum is a potential channel for updating WM, which is critical for memory and attention (Alhassen et al., 2023). Recently, it has been shown that a dual *n*-back WM training paradigm that includes auditory and visual stimuli can improve WM updating, which depends on striatal activation (Salminen et al., 2016). Current, whether WM training produces transfer effects is still controversial (Schwaighofer et al., 2015; Waris et al., 2015; Melby-Lervåg et al., 2016; Ripp et al., 2022).

Tai Chi is an ancient, chronic, low-intensity meditative exercise (Lan et al., 2008). Tai Chi practice can help players achieve calmness by coordinating prescribed movement patterns with mental concentration and slow breathing (Ainsworth et al., 2000). The intensity of Tai Chi does not exceed 55% of maximal oxygen intake (Li et al., 2001), besides, high levels of balance control and high levels of attention are required during Tai Chi practice (Melzer and Oddsson, 2004). In turn, the calmness and relaxation during Tai Chi practice can promote memory function (La Forge, 1997). Yau and Packer’s (2002) study concluded that Tai Chi resulted in improved cognitive function, including memory, attention span, mental alertness, and problem-solving ability. Kim’s et al. (2016) study found a moderately strong positive correlation (*r* = 0.41) between years of Tai Chi practice and nonverbal reasoning ability. Taylor-Piliae et al. (2010) considered Tai Chi practice as a backward digit span task, which develops endogenous attention and thus improves WM and complex executive cognitive processes (Kim et al., 2016). Cross-sectional studies and randomized controlled trials conducted in Hong Kong and the United States found that older adults who practiced Tai Chi had significantly better attention and memory than those who practiced exercises such as stretching or dancing (Chan et al., 2005; Man et al., 2010; Taylor-Piliae et al., 2010; Lam et al., 2011; Foster, 2013; Kattenstroth et al., 2013). Therefore it is believed that repeated Tai Chi practice may enhance specific cognitive functions (attention and memory) in Tai Chi practitioners.

A large number of studies support the association between mindfulness and well-being (Greeson, 2009). Mindfulness has been able to promote individuals’ mood (Khusid and Vythilingam, 2016), well-being and emotional balance (Goyal et al., 2014), and to reduce stress reactions (Pace et al., 2009), negative emotions (Khusid and Vythilingam, 2016), and the emotional responses to negative stimuli (Rosenberg et al., 2015), enhancing attention orientation and individuals’ ability to regulate emotions (Jha et al., 2007). In conclusion, the current research demonstrates that mindfulness intervention can affect various aspects of emotional processing, such as emotional intensity, emotional memory and emotional attentional bias (Wu et al., 2019). Yang short-form style of Tai Chi includes breathing relaxation, which differs from simple action exercises. It requires the practitioner to breathe slowly and softly, long breaths, concentrate greatly and relax all over the body during the exercise. The whole process has many similarities to mindfulness movement. Mindfulness is usually practiced through meditation, and the core of Tai Chi is paying attention to the regulation of body postures, breath, and awareness, which is similar to mindfulness meditation and is considered to a physical practice of mindfulness meditation (Jahnke et al., 2010). Some studies believed that mindfulness can be improved through body practice, such as Tai Chi, yoga, and other intentional physical exercises (Forge, 2005; Larkey et al., 2009). Caldwell et al. (2010) proved that Tai Chi practice could increase college students’ mindfulness, sleep quality, mood and perceived stress. A recent study has also demonstrated that autonomic nervous system (ANS) activities such as Baduanjin can effectively induce a state of calmness, characterized by high levels of calmness, perceived body activation, increased subjective vitality, attention focus, and body awareness, accompanied by pleasurable body sensations. At the physiological level, parasympathetic modulation (lnHF, RMSSD) was significantly decreased, whereas overall modulation (SDNN) was significantly increased, which indicated that autonomic nervous system (ANS) activities such as Tai Chi and Baduanjin can significantly increase individuals’ level of active regulation. More and more clinical studies have proved that Tai Chi, as an effective and safe therapeutic modality, not only copes with health problems, but also influences individuals’ general stress management (Wayne and Kaptchuk, 2008; Jahnke et al., 2010) and emotion regulation.

Some researchers argue that although WM primarily affects cognitive systems (Gathercole et al., 2016), nervous system will also make some necessary changes to sustain these changes, resulting in new cognitive mechanisms (Klingberg, 2010). Research has shown that WM training decreases activities in frontal–parietal brain networks and increases activity in subcortical areas (Dahlin et al., 2009). In addition, data also suggest that the hippocampus and left inferior frontal gyrus (LIFG) are involved in information retrieval from WM (Öztekin et al., 2009). In contrast, the θ oscillations in the medial prefrontal cortex are modulated by spatial WM and synchronized with the hippocampus *via* its ventral subregion (O'Neill et al., 2013). Thus, the prefrontal cortex and hippocampus are involved in WM. The strength of hippocampal-prefrontal synchrony correlates with behavioral performance. A study of the association between brain plasticity and Tai Chi practice demonstrated that cortical thickness in several key brain regions (e.g., prefrontal cortex) was significantly different in Tai Chi practitioners compared with individuals in control group (Wei et al., 2013). The result correlates with functional connectivity between the prefrontal cortex and key regions associated with emotion, as well as gray matter in the prefrontal cortex (Wei et al., 2016). Also, regular Tai Chi practice resulted in higher gray matter density in the inferior temporal and medial regions (including the hippocampus). Therefore, long-term Tai Chi practice may improve individuals’ memory by remodeling the structure and function of the hippocampus (Erickson et al., 2011; Yue et al., 2020). Temporal lobe structures (especially the hippocampus) highly influence individuals’ ability to remember situations in spatial and temporal contexts, specifically, the ability of the integration and retrieval of information (Grilli et al., 2018). Based on the available findings, it is believed that Tai Chi exercise alters several key brain regions in individuals, such as the prefrontal cortex and the inferior and medial temporal regions, including the hippocampus. And these regions are the same neurobiological regions of action produced by WM training. Therefore, we hypothesize that both Tai Chi practice and WM training may activate the prefrontal cortex’s role as a flexible hub in regulating individuals’ emotional well-being and enhance individuals’ temporal lobe structures (especially the hippocampus), thereby improving the individuals’ visual WM for situations, which in turn affects emotional regulation.

Taken together, The WM and emotion regulation are associated (Ribeiro et al., 2019). A WM in the area of cognition is particularly susceptible to emotion. The WM is crucial to modulating the efficacy of emotion regulation (Pe et al., 2013). Meanwhile, studies have proved that Tai Chi may positively impact attention, memory, etc. domains of cognitive function. The benefits of Tai Chi have been studied by many researchers in recent years. However, the previous studies focus mostly on the effect of Tai Chi intervention attention (Kim et al., 2016; Converse et al., 2020; Zhang et al., 2022), memory (Tao et al., 2016; Yue et al., 2020) and mood disorders (Wang et al., 2014; Kong et al., 2019), while few works have attempted to demonstrates Tai Chi experience can improve WMC and emotion regulation ability, and reveal the relationships between Tai Chi, WMC, and emotion regulation ability (Yeung et al., 2018). The current report aimed to explore this issue.

Based on the discussion above, we believe that Tai Chi exercise can improve individual WMC, which is greatly associated with emotional regulation. In this study, we hypothesize that it is possible to increase WMC through Tai Chi exercise, thereby improving an individual’s emotion regulation ability. The effect of Tai Chi practice on the facilitation of WM and emotion regulation can be assessed through the changes before and after Tai Chi practice.

## Materials and methods

### Sample size

The sample size was calculated with G*Power 3.1.9.7. *A priori* analysis was made by *F*-test (ANOVA) with the following input parameters: alpha = 0.05, power 1-beta = 0.80, effect size *f* = 0.25 (Cohen, 1988), resulting in a total sample size of 66 participants, with 33 participants per group (Faul et al., 2009). To allow for loss to follow-up and poor attendance, 60 participants were recruited as the initial sample.

### Participants

Sixty undergraduate students were recruited from a local university. Five participants quit in the middle of the experiment, so the rest 55 students were randomly divided into two groups. The mean age of the Tai Chi group was 20.17 ± 1.66 years (28 participants, 12 males, and 16 females). The mean age of the control group was 19.93 ± 1.74 years (27 participants, 13 males, and 14 females). All participants are students on campus, who have received about 13-year-education, without any practice in Tai Chi, Baduanjin, yoga, and other cognitive training before.

All participants were right-handed, had a normal or corrected-to-normal vision, had no reported psychiatric disorders, and did not consume alcohol, smoke, or any psychostimulants. Participants completed the Chinese Beck Anxiety Inventory (BAI) and the Beck Depression Inventory (BDI) before the trial. Participants in both groups did not show clinically significant symptoms of anxiety or depression. Participants signed informed consent forms before the experiment and paid for their participation. The study procedures were approved by the Institutional Review Board of the host university, and all procedures of this study were conducted in accordance with the principles of the Declaration of Helsinki.

### Procedure

The participants in the Tai Chi group learned Yang short-form style of Tai Chi (24 steps). This style of Tai Chi combines soft, large, slow, and open movements. Participants were offered Tai Chi classes three times a week for 12 consecutive weeks. A professional Tai Chi instructor taught all participants Yang short-form style of Tai Chi. Each class lasts 60 min, with a 15–20 min warming-up exercise (preparation stage) followed by a Tai Chi practice (40–45 min). The first stage is warming-up exercises (preparation stage), consisting of running, game playing, and rope skipping. The second stage is Tai Chi practice. Besides, all participants in the Tai Chi group were asked to practice Yang short-form style of Tai Chi independently for 30 min every day. The participants in the control group also did sports at the same time, in a different venue with 15–20 min warming-up of exercise (preparation stage) and 40–45 min of autonomous activities. The exercise content and intensity of the first stage were the same as those of the Tai Chi group. The second stage of autonomous activities consisted of jogging, brisk walk, and walks.

To equalize training intensity between groups in the exercise, the Polar RS800CXSD was used to monitor exercise intensity of Tai Chi and control group (4 students each group, 2 boys, and 2 girls), in order to make the participants in control group keep the same exercise intensity as those in Tai Chi group. In the first phase, the exercise load for both the Tai Chi and control groups was chosen to be a moderate exercise intensity of 60–69% of maximum heart rate (Delecluse et al., 2003). In the second phase, both the Tai Chi group and the control group were selected to exercise at a reserve heart rate of 57.8% (Lan et al., 2008), the control group exercise at the same level as Tai Chi at a moderate intensity. After 12 weeks, a *t*-test was conducted separately for the average heart rates of all participants in the first stage and the second stage, and there was no significant difference between the Tai Chi and the control groups (*p* > 0.05). It indicated that there was no difference in exercise intensity between the control group and the Tai Chi group during the intervention. The intensity of exercise of the two groups was consistent, and the exercise intensity monitoring was effective.

**Figure 1:**
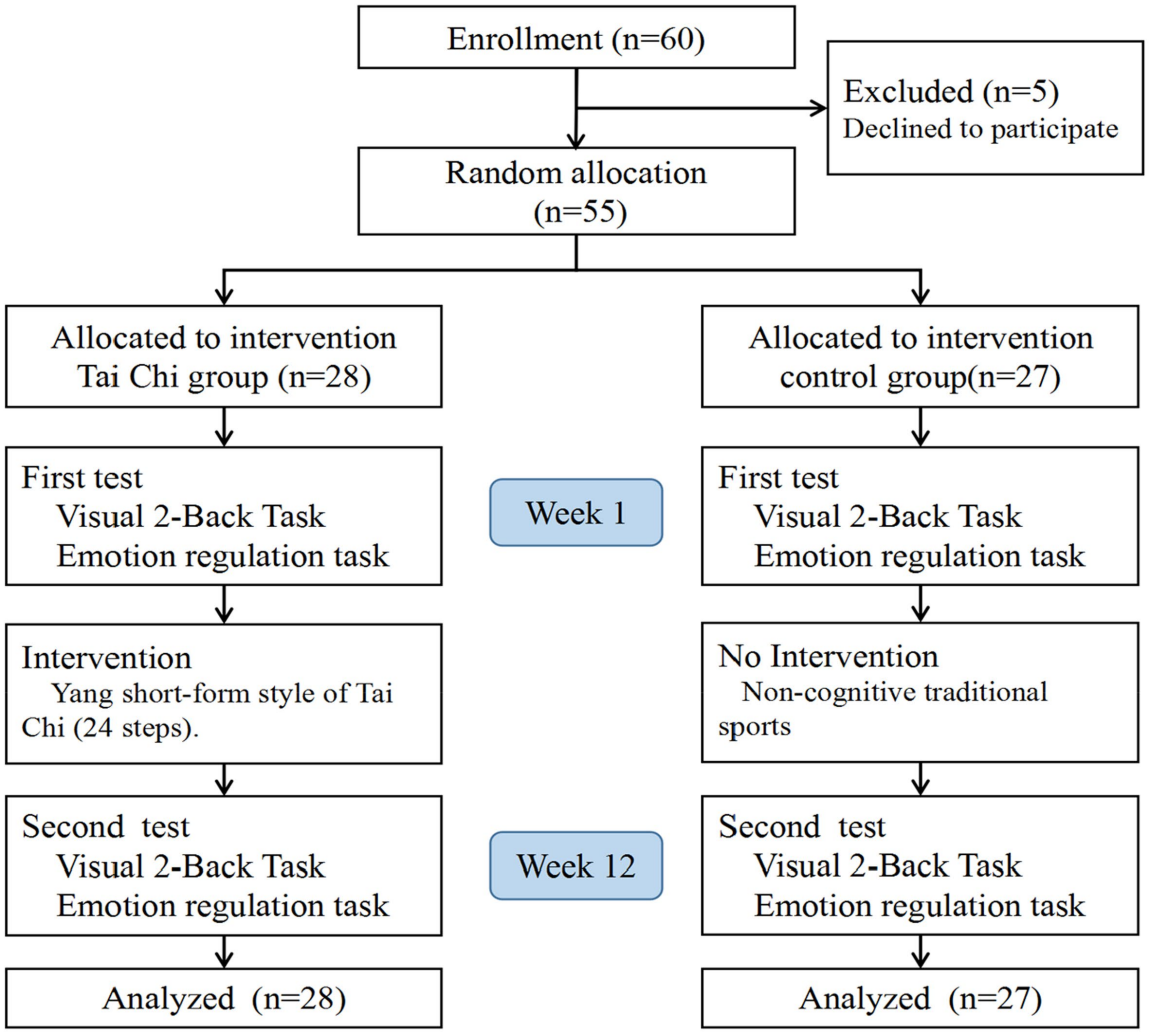
Flowchart of enrollment, baseline, and post-assessment.

During the 12-week practice, all the participants were asked not to do any cognitive exercises (such as yoga and Baduanjin) except for the target training, since those cognitive exercises contain positive meditation exercises that may improve the attention process (Sumantry and Stewart, 2021). While computer games were not allowed either, to block possible effects of computer games on participants’ fluid intelligence, WM, and other cognitive functions (Kokkinakis et al., 2017). All participants completed the visual 2-back task and emotion regulation task before (pre) and after (post) the 12-week program. Procedures are detailed in Figure 1.

#### Visual 2-back task

We hypothesized that Tai Chi would improve some form of kinesthetic/physical memory, which would help people perform better on tasks requiring postures. As participants internalized some postures by paying attention to or being aware of their bodies, they would be better able to update and recall visualizations of body movements. We selected a total of 30 sports action graphics, 10 (33%) of which were of Tai Chi actions, 10 (33%) of aerobic actions practiced by the control group, and 10 (33%) of unconventional sports actions (e.g., skating, boxing, weightlifting, etc.), three different types of action images to reduce bias. The 30 action images were also simplified into stick figures, and to reduce the influence of color factors, all graphics were in black and white, forming 30 action stick figures without color stimulus.

**Figure 2:**
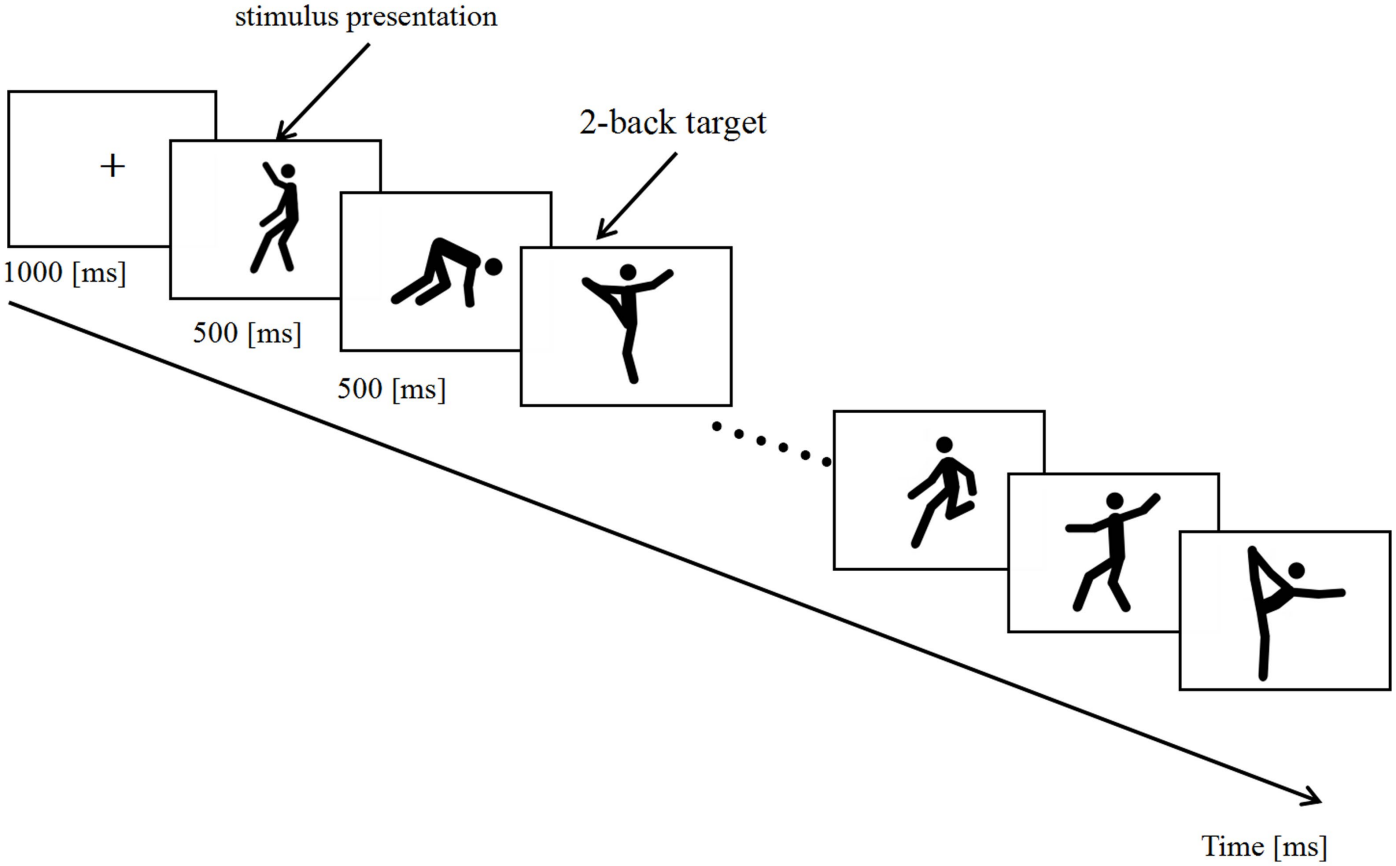
Trial structure of the Visual 2-back task (task 1).

The experiment was conducted using E-Prime 3.0.3.60 (E-Studio 3.0.3.43) software (Psychology Software Tools, Inc., Pittsburgh, PA, United States) and runs on a 19-inch monitor (refresh rate 60 Hz, resolution 1,920 × 1,080). Participants were seated in comfortable chairs in a quiet testing room, approximately 60 cm from the screen, and the stimuli were presented in the center of the screen. In the visual 2-back task, a series of picture stimuli consist of a schematic presentation of human actions in succession. A total of 30 stimuli were presented, each for 500 ms, followed by a 3,000 ms interval. The whole process includes practice and formal testing. In the testing, the participant must decide whether the picture presented is the same as the one presented in 2-back positions in the sequence. Press the number “1” key if the presentation is a target, and press the number “2” key if it is not a target. Each stimulus requires a response. The Accuracy Rate (AR) and response time (RT) of the stimuli were kept as behavioral performance data. Figure 1 illustrates the experimental paradigm. The test structure is shown in Figure 2.

#### Emotion regulation task

Emotion elicitation using the Geneva Affective Picture Database (GAPED) (obtained from SWISS CENTER FOR AFFECTIVE SCIENCES).^1^ GAPED increases the usability of visual emotions through a larger image database (730 images), which encompasses a total of 730 negative, neutral and positive images. The pictures were rated according to valence, arousal, and the congruence of the represented scene with internal (moral) and external (legal) norms (Dan-Glauser and Scherer, 2011). GAOED is currently being used for an increasing number of studies related to emotion rating and come emotion elicitation (Thuillard and Dan-Glauser, 2017; Gatti et al., 2018; Kragel et al., 2019).

Examples of how to conduct these normative studies are given in Lang’s (2008) overview of the rating process in the “effective ratings of pictures and instruction manual”, where a total of 180 images (60 positive, 60 neutral, and 60 negative) were selected from the Geneva Affective Picture Database (GAPED). For 60 neutral images, the valence was 55.68 ± 3.51, and arousal was24.39 ± 6.69. For 60 positive images, the valence was 89.25 ± 2.83, and arousal was 20.71 ± 9.51. For 60 negative images, the valence was 39.29 ± 4.02, and arousal was 52.68 ± 10.86. Each of the 60 positive, neutral and negative images were randomly divided into two sections of 30 images each, 30 images for a pre-test of emotion and 30 images for a post-test of emotion. A *t*-test was performed between the two sections, and the results showed that there was no difference among all conditions (All results had *p* values greater than 0.05). In each emotion induction test, 30 neutral images were presented after the gaze followed by 30 positive ones, constituting a positive emotion induction. After the positive picture stimulus, the participants made the first emotional evaluation. Then, 30 neutral images were presented followed by 30 negative images, constituting a negative emotion induction. After a negative picture stimulus, the participants made the second emotional evaluation. Participants were asked to perform tasks of emotional response evaluation after viewing positive and negative emotion pictures separately.

Emotional responses were evaluated using the Self-Assessment Manikin (SAM). Mehrabian and Russell (1974) designed The Semantic Differential Scale, which includes three dimensions of pleasure, arousal, and dominance, based on Spannung’s theory, to rate emotional responses. SAM represent simple puppets showing facial expressions (valence scale) and body movements (arousal scale) that participants use to rate how “happy” or “unhappy” (valence) and how “calm” or “excited” (arousal scale) they felt. Because of SAM, experimental data are simplified, differences between languages and cultures are reduced, and the scale can be better applied to populations unfamiliar with languages (Suvak et al., 2011). Therefore, SAM is a nonverbal assessment of arousal and valence levels, a self-rating method for measuring transient affective states, and a simple, nonverbal method for rapidly assessing people’s reports of affective experiences (Höfling et al., 2020; Kramer et al., 2021). Emotional response assessments were performed in the Self-Assessment Manikin (SAM) to rate the affective dimensions of valence (top panel), arousal (middle panel), and dominance (bottom panel). Participants were asked to rate their levels of valence, arousal, and self-control on a scale of 1–9 (for valence ratings, “1” was the most negative, and “9” was the most positive; for arousal ratings, “1 “represents the calmest, and “9” represents the most stimulating; for self-control, “1” represents complete control, and “9” represents no control).

**Figure 3:**
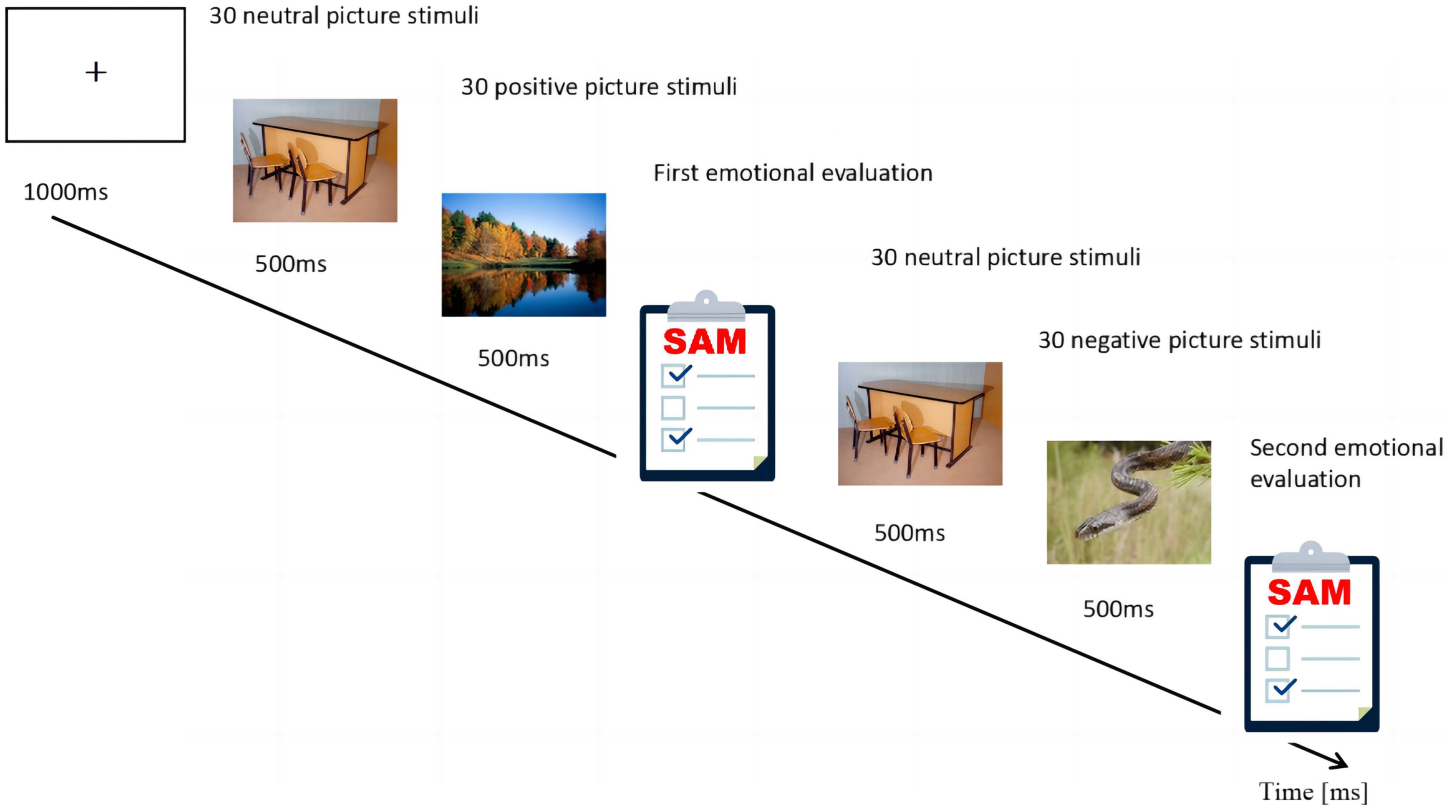
Trial structure of the emotion regulation task (Task 2). “Pictures for emotion induction” by The Geneva Affective Picture Database (GAPED) Reproduced with permission from Dan-Glauser and Scherer (2011).

In this study, we mainly focused on changes in emotion regulation ability. We use the difference (negative stimulus–response value minus positive stimulus–response value) between the three components (valence, arousal, and dominance) after viewing the positive and negative pictures to indicate the individuals’ emotional fluctuations and regulation ability. The test structure is shown in Figure 3.

#### Data processing procedure

We used E-Prime 3.0 software to extract response time (RT) and accuracy rate (AR) of Visual 2-Back Task, After displaying visual stimuli, a correct button press within 100–2,000 ms was regarded as an accurate response. Participant data entry in emotion regulation task was performed using a two-person input method. Then the value of the difference between emotional evaluation of positive stimuli and emotional evaluation of negative stimuli was calculated. Data from the visual 2-back task and the emotion regulation task were also merged in the processing.

Data were analyzed using SPSS v.25 (IBM Corp, Armonk, NY, United States). Participant demographic data such as age, height and weight and pretest data were analyzed by *t*-test. Shapiro and Wilk (1965) tests were performed to analyze distributional characteristics. Since there were only two groups, the sphericity test was not met. Greenhouse and Geisser (1959) correction was made when the assumption of sphericity was violated. To evaluate training effects (pre−/post-training) as within-subject factors and grouping (Tai Chi/Control Group) as a between-subject factor, a repeated measures general linear model (GLM) analysis was conducted. Training effects, training × group interaction effects, and between group effects are reported. Followed by Paired comparisons were conducted within each factor using Bonferroni corrections. In order to analyze significant interaction effects, post-hoc Bonferroni was used to determine main effects and simple main effects were analyzed to determine if there had been a significant interaction. We considered *p* values<0.05 as statistically significant (Table 1).

**Table 1:** Age, height, weight, and BMI of the participants in Tai Chi group and control group.

	Tai Chi group (*n* = 28)		Control group (*n* = 27)		*T*-Test	
	Mean	Std.	Mean	Std.	*T*	Sig. (2-tailed)
Age (years)	20.18	1.701	19.93	1.74	0.553	0.582
Height (cm)	159.79	4.533	160.04	4.519	−0.206	0.838
Weight (kg)	58.64	4.457	59.93	4.673	−1.042	0.302
BMI (kg/m^2^)	22.99	1.91	23.41	1.73	−0.835	0.407

BMI, Body-Mass-Index.

## Results

### Visual memory capability

Shapiro–Wilk test was used to examine the distribution of the data and it was found that Tai Chi Group AR-post (*W* = 0.967, *p* = 0.518), Control Group AR-post (*W* = 0.961, *p* = 0.3928) are consistent with the normal distributions of variables. Box’s test of equality of covariance matrices was not significant. Since there were only two groups, the sphericity test was not met. Greenhouse and Geisser (1959) correction was made when the assumption of sphericity was violated.

The AR repeated measures ANOVA test results indicated that all effects reached the significant level, time (*F*(1,53) 98.662, *p* ≤ 0.001, partial Eta square (*η*^2^) =0.651), group (*F*(1,53) 21.435, *p* ≤ 0.001, partial Eta square (*η*^2^) =0.288), interaction (groups × time; *F*(1,53) 50.815, *p* ≤ 0.001, partial Eta square (*η*^2^) =0.489), followed by significant interactions with analyses of simple main effects. Paired comparisons were conducted within each factor using Bonferroni corrections. The results showed that the mean value of the post-test in the Tai Chi group was higher than the mean value of the pre-test, and the differences were statistically significant (*F*(1,53) 148.240, *p* ≤ 0.001). In the comparison of the post-test results, the means of the Tai Chi group were higher than those of the control group, the differences were statistically significant (*F*(1,53) 54.899, *p* ≤ 0.001). The results are shown in Table 2.

**Table 2:** Results of repeated measures ANOVA^c^ for accuracy rate of working memory capability (M ± SD).

Group	AR-pre	AR-post	*F*	*p*	*η* ^2^
Tai Chi group (*n* = 28)	0.44 ± 0.07^a^	0.65 ± 0.06^a,b^	148.240	<0.001	0.737
Control group (*n* = 27)	0.45 ± 0.08	0.48 ± 0.11^b^	3.862	0.054	0.068
*F*	0.028	54.899			
*p*	0.867	<0.001			
*η* ^2^	0.001	0.509			

*F*_time_ = 98.662, *P*_time_ < 0.001, *η*^2^ = 0.651, *F*_group_ = 21.435, *P*_group_ < 0.001, *η*^2^ = 0.288, *F*_time*group_ = 50.815, time*group < 0.001, *η*^2^ = 0.489. AR Visual 2-back task accuracy rate. ^c^Greenhouse and Geisser (1959) corrections were applied. ^a,b^Means with different superscripts differ significantly at *p* < 0.05 using Bonferroni paired comparisons.

**Figure 4:**
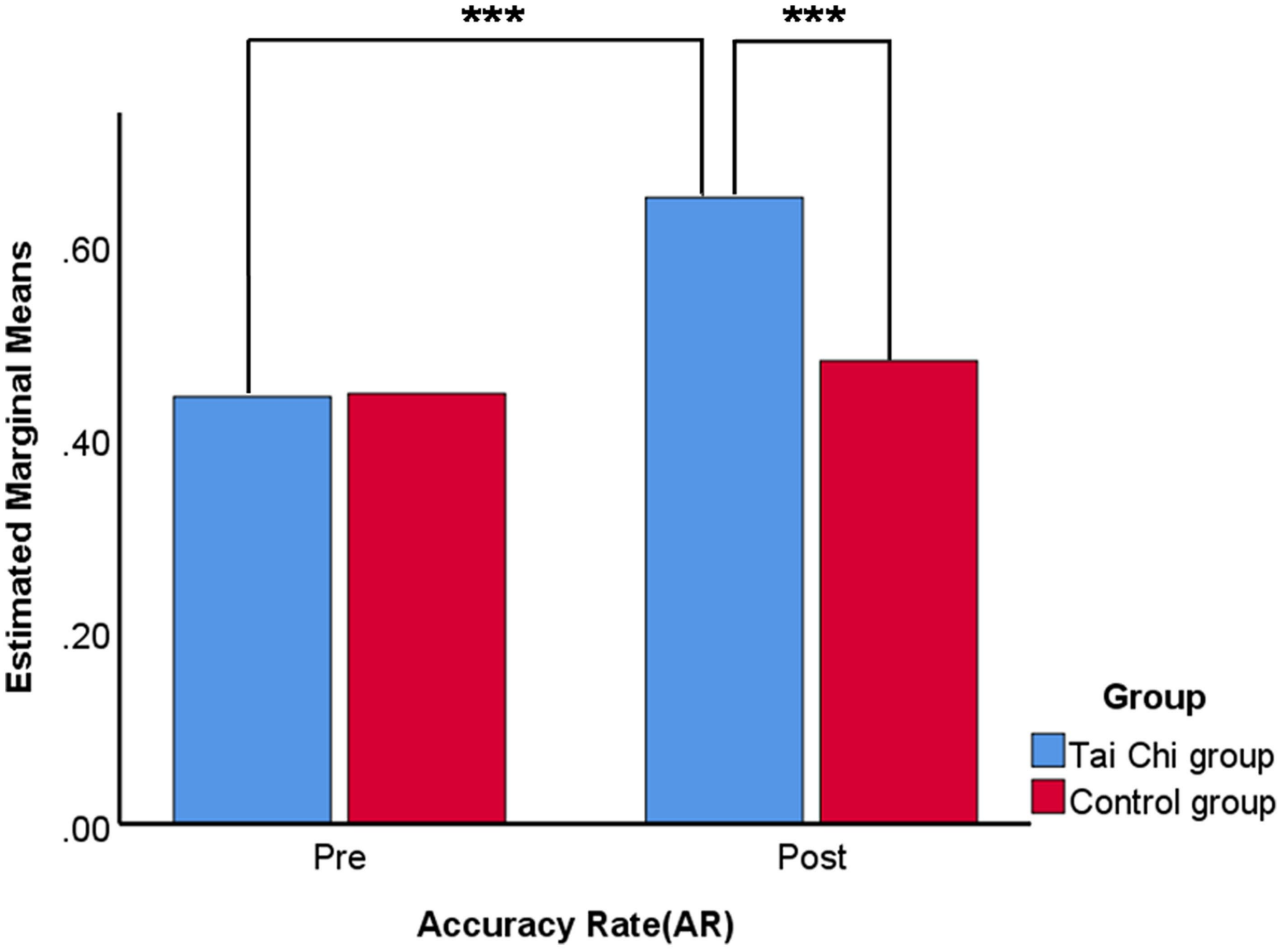
Paired comparison of Accuracy Rate (AR) on visual working memory tests. Bonferroni adjustment for multiple comparisons. ***p-value <0.001.

Paired comparison of Accuracy Rate (AR) on visual WM tests showed that, after 12 weeks, the Tai Chi group had a significant improvement in the accuracy rate in the Visual 2-Back Task, indicating that their visual memory capability was improved in the Tai Chi group, after 12 weeks of Tai Chi practice. The results are shown in Figure 4.

Shapiro–Wilk test was used to examine the distribution of the data and it was found that Tai Chi Group RT-post (*W* = 0.970, *p* = 0.575), Control Group RT-post (*W* = 0.961, *p* = 0.367), are consistent with the normal distributions of variables. Box’s test of equality of covariance matrices was not significant. Since there were only two groups, the sphericity test was not met. Greenhouse and Geisser (1959) correction was made when the assumption of sphericity was violated.

The RT repeated measures ANOVA test results indicated all effects reached the significant level too, time (*F*(1,53) 67.211, *p* ≤ 0.000, partial Eta square (*η*^2^) = 0.559), group (*F*(1,53) 45.685, *p* ≤ 0.001, partial Eta square (*η*^2^) = 0.463), interaction (groups × time; *F*(1,53) 79.526, *p* ≤ 0.001, partial Eta square (*η*^2^) =0.600), followed by Significant interactions with analyses of simple main effects. Paired comparisons were conducted within each factor using Bonferroni corrections. The results showed that the mean value of the post-test in the Tai Chi group was lower than the mean value of the pre-test, and the differences were statistically significant (*F*(1,53) 149.191, *p* ≤ 0.001). In the comparison of the post-test results, the means of the Tai Chi group were lower than those of the control group, the differences were statistically significant (*F*(1,53) 99.454, *p* ≤ 0.001). The results are shown in Table 3.

**Table 3:** Results of repeated measures ANOVA^c^ for response time of working memory capability (M ± SD).

Group	RT-pre	RT-post	*F*	*p*	*η* ^2^
Tai Chi group (*n* = 28)	1,006.08 ± 39.82^a^	862.92 ± 65.33^a,b^	149.191	<0.001	0.738
Control group (*n* = 27)	1,000.49 ± 48.01	1,006.51 ± 37.11^b^	0.254	0.616	0.005
*F*	0.222	99.454			
*p*	0.64	<0.001			
*η* ^2^	0.004	0.652			

*F*_time_ = 67.210, *P*_time_ < 0.001, *η*^2^ = 0.559, *F*_group_ = 45.685, *P*_group_ < 0.001, *η*^2^ = 0.463, *F*_time*group_ = 79.526, *P*_time*group_ < 0.001, *η*^2^ = 0.600.

RT Visual 2-back task reaction time. ^c^Greenhouse and Geisser (1959) corrections were applied. ^a,b^Means with different superscripts differ significantly at *p* < 0.05 using Bonferroni paired comparisons.

**Figure 5:**
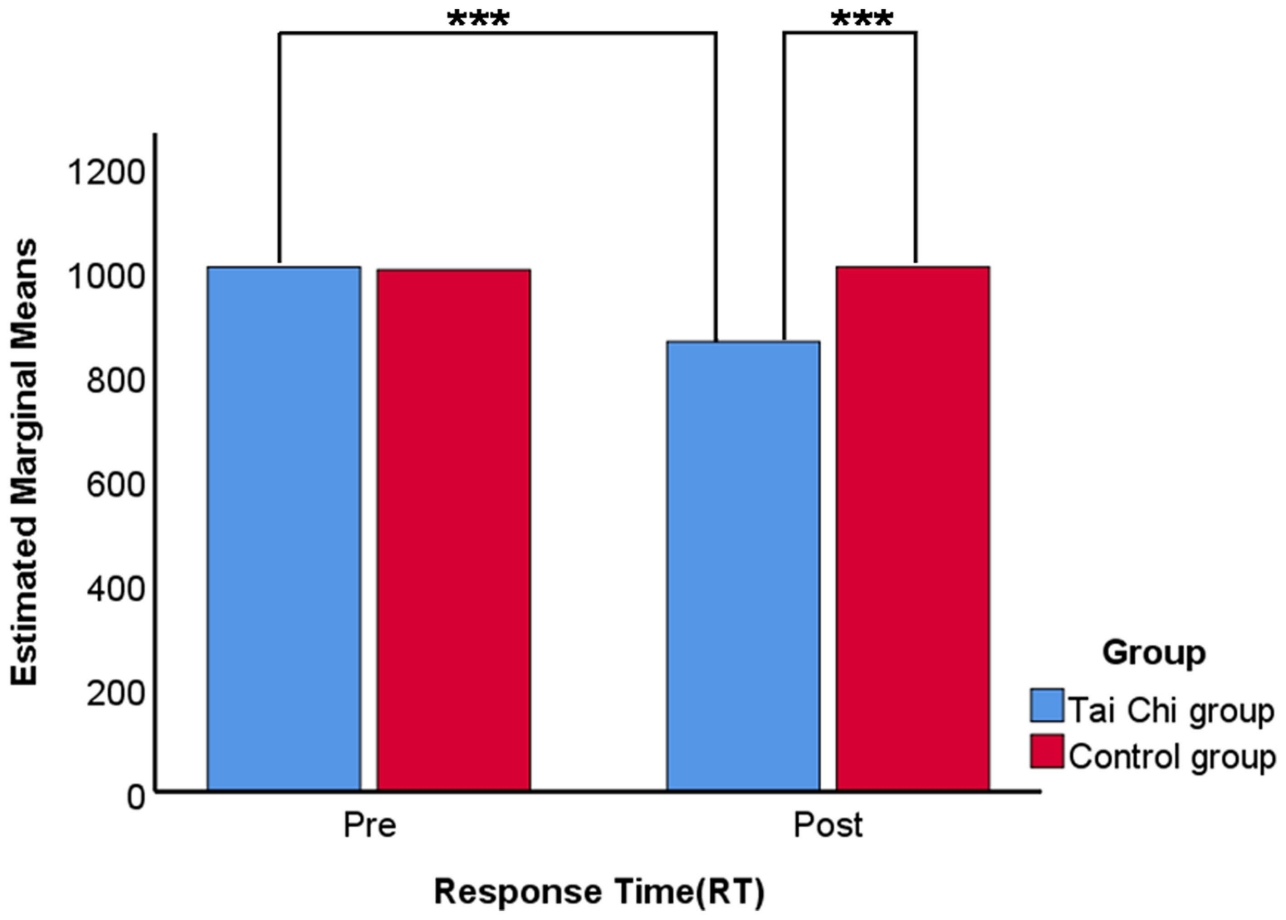
Paired comparison of Response Time (RT) on visual working memory tests. Bonferroni adjustment for multiple comparisons. ***p-value <0.001.

Paired comparison of Response Time (RT) on visual WM tests showed that, after 12 weeks, the Tai Chi group had a significant decrease in reaction time in the Visual 2-Back Task, indicating that their visual memory capability was improved in the Tai Chi group, after 12 weeks of Tai Chi practice. The results are shown in Figure 5.

### Emotion regulation ability

Shapiro–Wilk test was used to examine the distribution of the data and it was found that Tai Chi Group Valence Difference-post (*W* = 0.965, *p* = 0.459), Control Group Valence Difference-post (*W* = 0.943, *p* = 0.144) are consistent with the normal distributions of variables. Box’s test of equality of covariance matrices was not significant (*F* = 0.355, *p* = 0.785). Since there were only two groups, the sphericity test was not met. Greenhouse and Geisser (1959) correction was made when the assumption of sphericity was violated.

Repeated Measures ANOVA of the valence differences of the Tai Chi group and the control group revealed that all effects reached the significant level, time (*F* = 7.28, *p* < 0.01, partial Eta square (*η*^2^) =0.121), group (*F* = 4.16, *p* < 0.05, partial Eta square (*η*^2^) =0.073), time*group (*F* = 10.16, *p* < 0.01, partial Eta square (*η*^2^) =0.161), followed by significant interactions with analyses of simple main effects. Paired comparisons were conducted within each factor using Bonferroni corrections. The results showed that the mean value of the post-test in the Tai Chi group was lower than the mean value of the pre-test, and the differences were statistically significant (*F*(1,53) 17.649, *p* ≤ 0.001). In the comparison of the post-test results, the means of the Tai Chi group were lower than those of the control group, the differences were statistically significant (*F*(1,53) 11.497, *p* ≤ 0.001). The results are shown in Table 4.

**Table 4:** Results of covariance and repeated measures ANOVA^c^ for valence difference (M ± SD).

Group	Pre	Post	*F*	*p*	*η* ^2^
Tai Chi group (*n* = 28)	3.14 ± 1.35^a^	1.36 ± 1.74^ab^	17.649	<0.001	0.250
Control group (*n* = 27)	2.81 ± 1.57	2.96 ± 1.76^b^	0.117	0.734	0.002
*F*	0.691	11.497			
*P*	0.410	<0.001			
*η* ^2^	0.013	0.178			

*F*_time_ = 7.286, *P*_time_ < 0.01, *η*^2^ = 0.121, *F*_group_ = 4.164, *P*_group_ < 0.05, *η*^2^ = 0.073, *F*_time*group_ = 10.161, *P*_time*group_ < 0.01, *η*^2^ = 0.161. ^c^Greenhouse and Geisser (1959) corrections were applied. ^a,b^Means with different superscripts differ significantly at *p* < 0.05 using Bonferroni paired comparisons.

**Figure 6:**
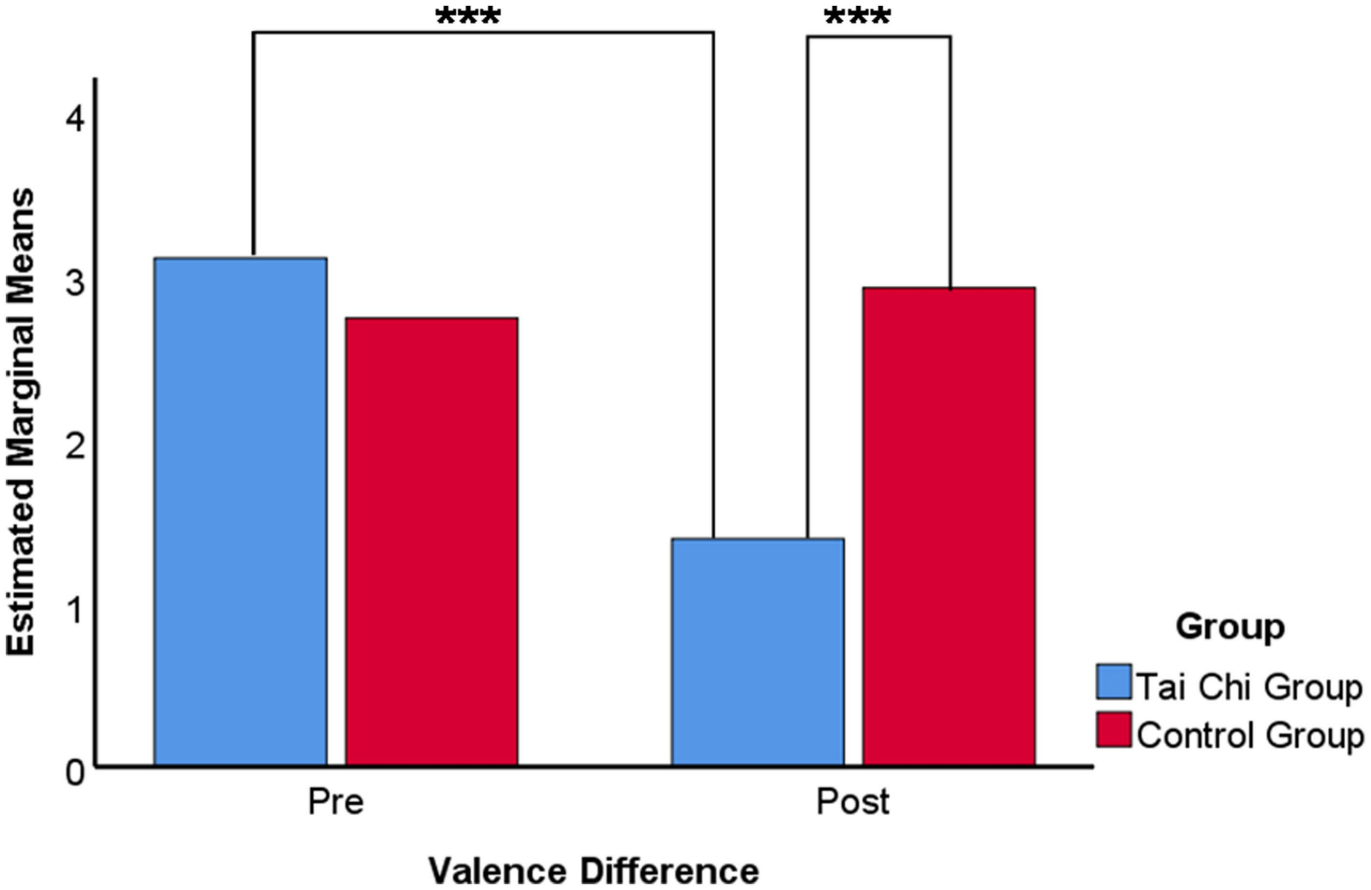
Multiple means comparison of valence difference on emotion regulation ability. Bonferroni adjustment for multiple comparisons. ***p-value <0.001, **p-value <0.01, *p-value <0.05.

Paired comparisons were conducted within each factorusing Bonferroni corrections, indicating that the mean score of valence difference for the Tai Chi group (*M* = 1.36, SD = 1.74) was significantly lower than that for the control group (*M* = 2.96, SD = 1.76). The difference was statistically significant (*p* < 0.001). Also the results of the pre-test (*M* = 3.14, SD = 1.35) with the Tai Chi group were significantly lower, with a statistically significant difference (*p* < 0.001). The results are shown in Figure 6.

Shapiro–Wilk test was used to examine the distribution of the data and it was found that Tai Chi Group Arousal Difference-post (*W* = 0.968, *p* = 0.459), Control Group Arousal Difference-post (*W* = 0.948, *p* = 0.192) are consistent with the normal distributions of variables. Box’s test of equality of covariance matrices was not significant (*F* = 1.121, *p* = 0.339). Since there were only two groups, the sphericity test was not met. Greenhouse and Geisser (1959) correction was made when the assumption of sphericity was violated.

Repeated Measures ANOVA of the arousal differences of the Tai Chi group and the control group revealed that all effects reached the significant level, time (*F* = 5.180, *p* < 0.05, partial Eta square (*η*^2^) =0.089), Group (*F* = 7.266, *p* < 0.01, partial Eta square (*η*^2^) =0.121), time*Group (*F* = 4.231, *p* < 0.05, partial Eta square (*η*^2^) =0.074), followed by significant interactions with analyses of simple main effects. Paired comparisons were conducted within each factor using Bonferroni corrections. The results showed that the mean value of the post-test in the Tai Chi group was lower than the mean value of the pre-test, and the differences were statistically significant (*F*(1,53) 9.56, *p* ≤ 0.01). In the comparison of the post-test results, the means of the Tai Chi group were lower than those of the control group, the differences were statistically significant (*F*(1,53) 10.17, *p* ≤ 0.01). The results are shown in Table 5.

**Table 5:** Results of repeated measures ANOVA^c^ for arousal difference (M ± SD).

Group	Pre	Post	*F*	*p*	*η* ^2^
Tai Chi group (*n* = 28)	3.04 ± 1.32^a^	1.57 ± 1.66^ab^	9.561	<0.01	0.153
Control group (*n* = 27)	3.11 ± 1.81	3.04 ± 1.74^b^	0.024	0.879	0.000
*F*	0.031	10.173			
*p*	0.860	<0.01			
*η* ^2^	0.001	0.161			

*F*_time_ = 5.180, *P*_time_ < 0.05, *η*^2^ = 0.089, *F*_group_ = 7.266, *P*_group_ < 0.01, *η*^2^ = 0.121, *F*_time*group_ = 4.231, *P*_time*group_ < 0.05, *η*^2^ = 0.074. ^c^Greenhouse and Geisser (1959) corrections were applied. ^a,b^Means with different superscripts differ significantly at *p* < 0.05 using Bonferroni paired comparisons.

**Figure 7:**
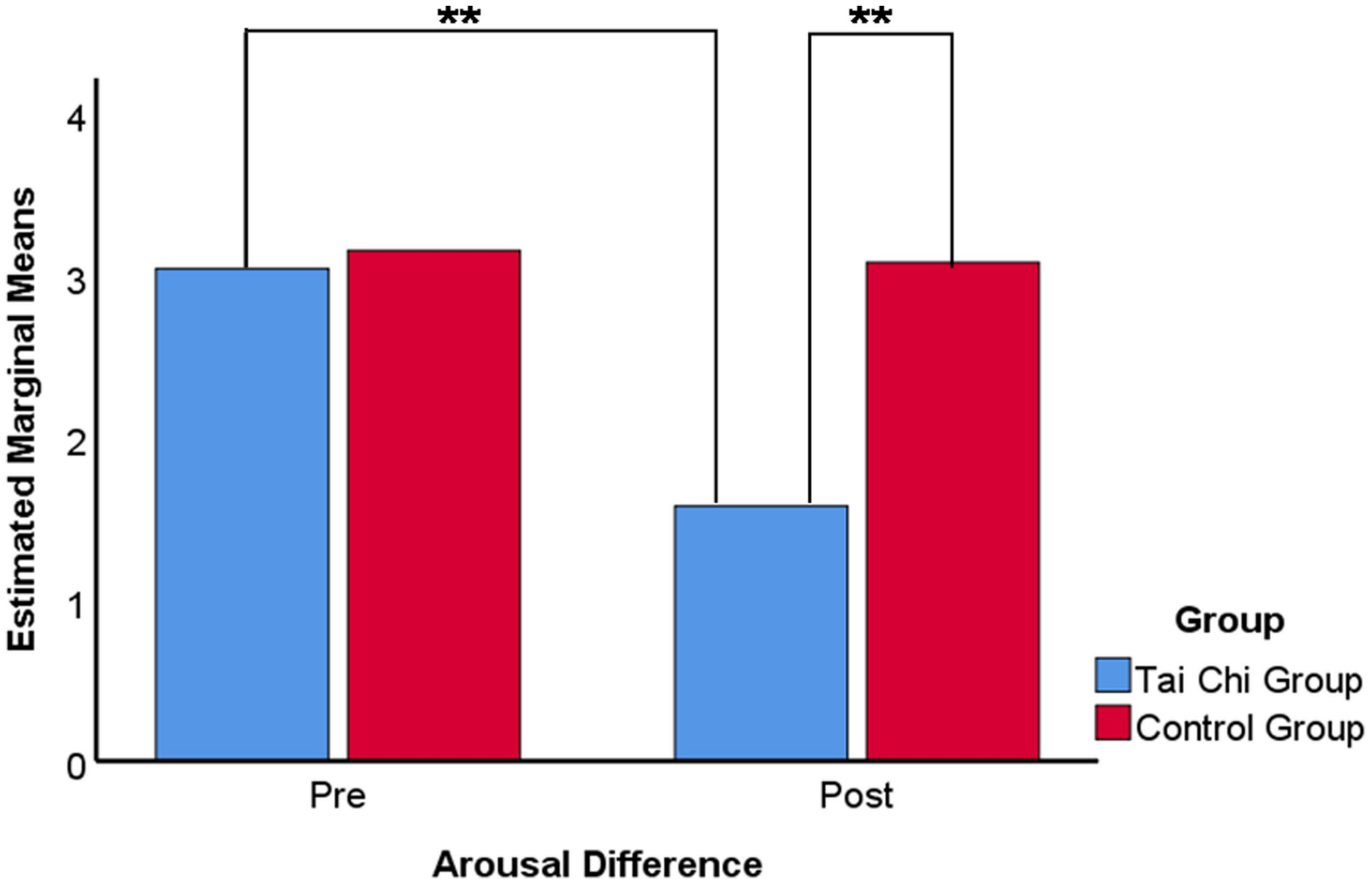
Multiple means comparison of arousal difference on emotion regulation ability. Bonferroni adjustment for multiple comparisons. ***p-value <0.001, **p-value <0.01, *p-value <0.05.

Paired comparisons were conducted within each factor using Bonferroni corrections, indicating that the mean score of arousal difference for the Tai Chi group (*M* = 1.57, SD = 1.66) was significantly lower than that for the control group (*M* = 3.04, SD = 0.17). The difference was statistically significant (*p* < 0.01). Also the results of the pre-test (*M* = 3.04, SD = 1.32) with the Tai Chi group were significantly lower, with a statistically significant difference (*p* < 0.01). The results are shown in Figure 7.

Shapiro–Wilk test was used to examine the distribution of the data and it was found that Tai Chi Group Dominance Difference-post (*W* = 0.938, *p* = 0.096), Control Group Dominance Difference-post (*W* = 0.935, *p* = 0.093) are consistent with the normal distributions of variables. Box’s test of equality of covariance matrices was not significant (*F* = 0.479, *p* = 0.697). Since there were only two groups, the sphericity test was not met. Greenhouse and Geisser (1959) correction was made when the assumption of sphericity was violated.

Repeated Measures ANOVA of the dominance differences of the Tai Chi group and the control group revealed that all effects reached the significant level, time (*F* = 7.921, *p* < 0.01, partial Eta square (*η*^2^) =0.130), group (*F* = 5.829, *p* < 0.05, partial Eta square (*η*^2^) =0.099), time*group (*F* = 10.269, *p* < 0.01, partial Eta square (*η*^2^) =0162), followed by Significant interactions with analyses of simple main effects. Paired comparisons were conducted within each factor using Bonferroni corrections. The results showed that the mean value of the post-test in the Tai Chi group was lower than the mean value of the pre-test, and the differences were statistically significant (*F* (1,53)18.45, *p* ≤ 0.001). In the comparison of the post-test results, the means of the Tai Chi group were lower than those of the control group, the differences were statistically significant (*F*(1,53) 13.30, *p* ≤ 0.001). The results are shown in Table 6.

**Table 6:** Results of repeated measures ANOVA^c^ for dominance difference (M ± SD).

Group	Pre	Post	*F*	*p*	*η* ^2^
Tai Chi group (*n* = 28)	3.14 ± 1.72^a^	1.43 ± 1.83^ab^	18.450	<0.001	0.258
Control group (*n* = 27)	3.07 ± 1.4	3.19 ± 1.73^b^	0.075	0.786	0.001
*F*	0.027	13.307			
*p*	0.870	<0.001			
*η* ^2^	0.001	0.201			

*F*_time_ = 7.921, *P*_time_ < 0.01, *η*^2^ = 0.130, *F*_group_ = 5.829, *P*_group_ < 0.05, *η*^2^ = 0.099, *F*_time*group_ = 10.269, *P*_time*group_ < 0.01, *η*^2^ = 0.162. ^c^Greenhouse and Geisser (1959) corrections were applied. ^a,b^Means with different superscripts differ significantly at *p* < 0.05 using Bonferroni paired comparisons.

**Figure 8:**
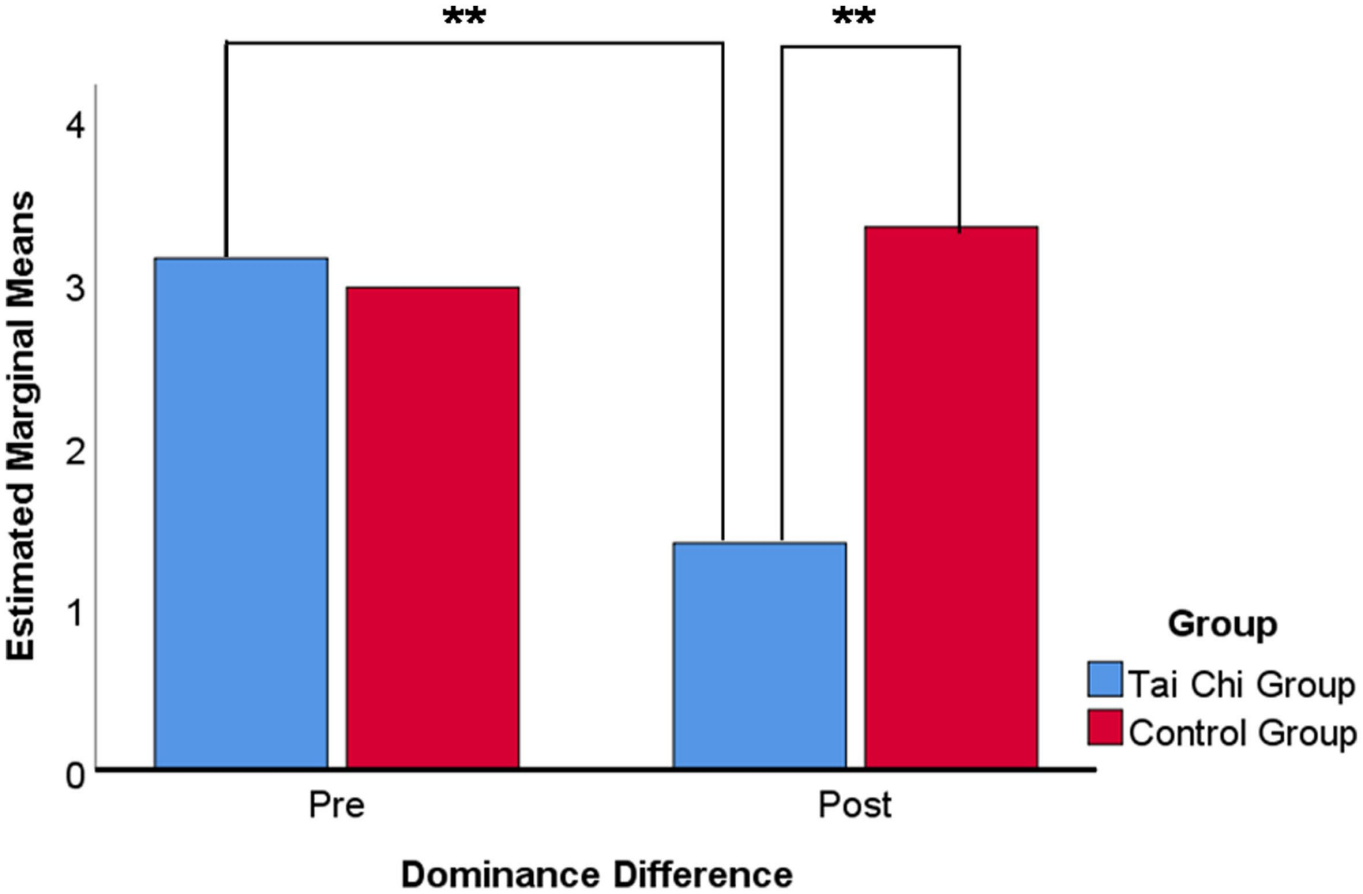
Multiple means comparison of dominance difference on emotion regulation ability. Bonferroni adjustment for multiple comparisons. ***p-value <0.001, **p-value <0.01, *p-value <0.05.

Paired comparisons were conducted within each factorusing Bonferroni corrections indicated that the mean score of dominance difference for the Tai Chi group (*M* = 1.43, SD = 1.83) was significantly lower than that for the control group (*M* = 3.19, SD = 1.73). The difference was statistically significant (*p* < 0.01). Also the results of the pre-test (*M* = 3.14, SD = 1.72) with the Tai Chi group were significantly lower, with a statistically significant difference (*p* < 0.01). The results are shown in Figure 8.

## Discussion

In the present study, we attempted to assess the facilitation effect of Tai Chi training on the visual working ability capacity and emotion regulation ability. The results showed: first, individuals’ Visual Memory Capacity has been improved after a 12-week Tai Chi training; second, the difference in emotional fluctuations between positive and negative stimuli has been narrowed, suggesting that Tai Chi promoted individuals’ emotion regulation. These findings proved that Tai Chi training can not only improve individual’s Visual Memory Capacity and enhance their emotion regulation, but decrease individual’s emotional sensitivity, which reflected individual’s stronger emotion regulation (Liu et al., 2020). Therefore, Tai Chi training can produce stronger emotion-regulation ability, which supporting the previous studies (Yeung et al., 2018).

Attention is an important tool and a key component in facilitating emotion regulation (Wadlinger and Isaacowitz, 2011). Although Tai Chi is just a slow action sequence practice, it requires complex body posture control and memory training for movement sequences (Miller and Taylor-Piliae, 2014). On one hand, the coordination between attention, action, postural control, and visual images in Tai Chi may provide additional cognitive stimulation. On the other hand, Tai Chi exercise requires attention to specific postures and action sequences, which enhances individuals’ visual attention (visual span; Lam et al., 2011). For example, When Tai Chi is practiced, the practitioner is first asked to relax, to be calm and gentle, to leave all distractions behind, and to focus all attention on their breathing and senses, which is a kind of attention training. Attention deployment is now considered to be one of the core mechanisms in emotion regulation (Boelens et al., 2021) and a prior emotion regulation strategy that has an important impact on the generation, maintenance, or modification of emotions. Compared with traditional sports, there is more training in attention control in Tai Chi practice.

Due to limited WMC, attention control is needed in attention selection and emotion regulation reassessment (Sheppes et al., 2014). WMC affects the selection and allocation of resources. However, orienting functions are attention network mechanisms that select and filter external information, including attention engagement, attention shifting, and attention disengagement (Moriya and Tanno, 2009). Therefore, within the framework of emotion regulation, information selection and filtering by orienting functions will be a perpetual component of WM training. Individuals with relatively high WMC can orient their attention to the objects requiring attention, whereas individuals with relatively low WMC have their attention in a more distracted manner (Xiu et al., 2018). The Tai Chi training process requires practitioners to focus and orient their attention on breathing, postures, action sequences, and internal experiences, meanwhile filtering out external information by attention engagement, attention shifting, and attention disengagement, which can improve the orienting function of individual attention. In conclusion, Tai Chi practice improves the individual’s WMC through visual WM of movements.

The broaden-and-build theory of positive emotions indicated that attention can be (re)directed. Positive emotions broaden attention in vision, allowing individuals to process more information (Rowe et al., 2007). Positive emotions expand visual attention, allowing individuals to process more information (Rowe et al., 2007), so positive emotions is related to an broadened attention breadth, whereas negative emotions is associated with a narrowed attention breadth. Thus, it is predicted that there is an association between emotional regulation (ER) and the visual attention breadth of emotional information (Fredrickson and Joiner, 2002). Similarly, there is a multifaceted relationship between distraction strategies in emotion regulation strategies and the visual attention breadth for emotional information (Boelens et al., 2021). Tai Chi practice makes individuals pay attention to body posture, action sequences, breathing, and strength at all times, allowing the individuals to increase attention breadth and improve their emotion regulation. However, some scholars have questioned the broaden-and-build theory of positive emotions (Bruyneel et al., 2013), arguing that there was no relevant evidence got (Rowe et al., 2007; Bendall and Thompson, 2015). In this regard, it has been argued that the relationship between emotions and attention breadth is influenced by individual differences due to individual personality traits. Maclean and Arnell’s (2010) research into the fast continuous presentation tasks suggested that neuroticism and extraversion (related to emotional responses) may reduce the effects of emotion on attention. Individuals with higher levels of extraversion and lower levels of neuroticism would have increased cognitive resources, which could improve their performance in the tasks of measuring attention. Such personality traits would interact with the effects of emotions (Bendall et al., 2021). Individuals with higher extroversion are slower to find changes, and focus more on the balance between speed and accuracy in task performance (Hahn et al., 2015).

Tai Chi is a slow, gentle movement, which requires the practitioner to be focused at all times during the practice, not only on the current actions, but on the internal experience, which is very similar to mindfulness practice. Long-term Tai Chi practice will enable individuals to have more resources for attention, although the process is very slow. Tai Chi practitioners need to have accurate judgment, the balance of postures and action sequences, so long-term Tai Chi practice will reduce individuals’ neuroticism, improve their visual WM, ultimately, to reduce individuals’ emotion fluctuation and improve their emotion regulation ability.

## Limitations

This was a randomized controlled trial for college students. For the characteristics of the Tai Chi exercise, it could not be guaranteed that all participants had high expectations for the task and devoted themselves to it. To be able to more effectively validate the positive effects of Tai Chi on WM and emotion regulation, future studies on this similar topic should be rigorous in screening participants. In the follow-up study, we need to set up 3 groups: A Tai Chi group, A sports Group and a meditation group with zero exercise to see if there’s an interaction.

Second, there are large individual differences in emotion regulation ability. Although we performed uniformity in demographic characteristics, age, and gender, and conducted screening for mental health disorders before the experiment, we still could not guarantee complete individual differences in each subject. Also, emotions are easily subject to external influences, despite our strict procedural controls, so we still cannot guarantee that individuals can completely avoid the impact of external events on emotions over a longer span of time, especially the effects of mobile phones and computer games on college students’ cognition and emotion. In future studies, we will use a more “objective” method to measure emotions, such as skin conductance, heart rate while viewing the emotionally negative images, or neuroimaging.

Thirdly, this study only included assessments of baseline and post-intervention, missing intermediate testing links. The number of tests will be increased in future studies to assess if such positive effects can persist after the study. Due to the close relationship between Tai Chi and attention control, future research will include Attention blink task and Flanker task.

Fourth, a large number of relevant studies have support the relationship between WM and emotion regulation ability, and the promoting effect of Tai Chi exercise on cognitive, the present study also proved the point that Tai Chi exercise can promote WM and emotion regulation. For the reason that the design of the study did not provide a good account of the causal conclusion between action memory and emotion regulation, the quasi-experimental design in this study limited the possibilities for causal conclusions. Therefore, the structural equation modeling approach can be considered in future research and design.

## Conclusion

The evidence from this study suggests that movement skills learning in Tai Chi can improve individuals’ Visual Memory Capacity and enhance their emotion regulation ability. The data support our speculation that action memory training in Tai Chi exercise may improve individuals’ WMC, and than improve emotion regulation ability, which has provided insightful information for customized exercise programs for emotion regulation in adolescents. Thus, we recommend those adolescents who are experiencing volatile moods and poor emotion regulation attend regular Tai Chi classes, which could contribute to their emotional health.

## Data availability statement

The raw data supporting the conclusions of this article will be made available by the authors, without undue reservation.

## Ethics statement

The studies involving human participants were reviewed and approved by the School of Physical Education, Weinan Normal University Academic Committee. The patients/participants provided their written informed consent to participate in this study.

## Author contributions

The data was collected in the field at Weinan Normal University, and then analyzed here. YW contributed to study design and formal analysis. QY contributed to data collection and investigation. JT contributed to writing–review, editing, and revising the manuscript. All authors approved the final manuscript and listed qualification for authorship and agreed with the order of authorship.

## Conflict of interest

The authors declare that the research was conducted in the absence of any commercial or financial relationships that could be construed as a potential conflict of interest.

## Publisher’s note

All claims expressed in this article are solely those of the authors and do not necessarily represent those of their affiliated organizations, or those of the publisher, the editors and the reviewers. Any product that may be evaluated in this article, or claim that may be made by its manufacturer, is not guaranteed or endorsed by the publisher.

## Footnotes

^1^ https://www.unige.ch/cisa/research/materials-and-online-research/research-material/
