# Supplementary material for: Tai Chi exercise improves working memory capacity and emotion regulation ability
Source: Front Psychol. 2023 Feb 17;14:1047544. doi: 10.3389/fpsyg.2023.1047544 (PMC9983368; doi:10.3389/fpsyg.2023.1047544)
Supplement: Supplementary file 1 [file Table_1.DOC]

Results of normality test of emotion regulation ability test


Case Processing Summary	
	Group	Cases	
		Valid	Missing	Total	
		N	Percent	N	Percent	N	Percent	
Valence Difference-pre	Tai Chi Group	28	100.0%	0	0.0%	28	100.0%	
	Control Group	27	100.0%	0	0.0%	27	100.0%	
Arousal Difference-pre	Tai Chi Group	28	100.0%	0	0.0%	28	100.0%	
	Control Group	27	100.0%	0	0.0%	27	100.0%	
Dominance Difference-pre	Tai Chi Group	28	100.0%	0	0.0%	28	100.0%	
	Control Group	27	100.0%	0	0.0%	27	100.0%	
Valence Difference-post	Tai Chi Group	28	100.0%	0	0.0%	28	100.0%	
	Control Group	27	100.0%	0	0.0%	27	100.0%	
Arousal Difference-post	Tai Chi Group	28	100.0%	0	0.0%	28	100.0%	
	Control Group	27	100.0%	0	0.0%	27	100.0%	
Dominance Difference-post	Tai Chi Group	28	100.0%	0	0.0%	28	100.0%	
	Control Group	27	100.0%	0	0.0%	27	100.0%	


Tests of Normality	
	Group	Kolmogorov-Smirnova	Shapiro-Wilk	
		Statistic	df	Sig.	Statistic	df	Sig.	
Valence Difference-pre	Tai Chi Group	.165	28	.048	.937	28	.090	
	Control Group	.217	27	.002	.934	27	.086	
Arousal Difference-pre	Tai Chi Group	.189	28	.011	.934	28	.077	
	Control Group	.133	27	.200*	.963	27	.442	
Dominance Difference-pre	Tai Chi Group	.145	28	.135	.937	28	.094	
	Control Group	.193	27	.011	.930	27	.067	
Valence Difference-post	Tai Chi Group	.144	28	.146	.965	28	.459	
	Control Group	.144	27	.155	.943	27	.144	
Arousal Difference-post	Tai Chi Group	.148	28	.117	.968	28	.516	
	Control Group	.158	27	.081	.948	27	.192	
Dominance Difference-post	Tai Chi Group	.235	28	.000	.938	28	.096	
	Control Group	.199	27	.007	.935	27	.093	
*. This is a lower bound of the true significance.	
a. Lilliefors Significance Correction	


Valence Difference-pre

Histograms


Normal Q-Q Plots


Arousal Difference-pre
Histograms


Normal Q-Q Plots


Dominance Difference-pre

Histograms


Normal Q-Q Plots


Valence Difference-post
Histograms


Normal Q-Q Plots


Arousal Difference-post

Histograms


Normal Q-Q Plots


Dominance Difference-post
Histograms


Normal Q-Q Plots
