# Supplementary material for: Tai Chi exercise improves working memory capacity and emotion regulation ability
Source: Front Psychol. 2023 Feb 17;14:1047544. doi: 10.3389/fpsyg.2023.1047544 (PMC9983368; doi:10.3389/fpsyg.2023.1047544)
Supplement: Supplementary file 2 [file Table_2.DOC]

Results of normality test of Visual Memory Capability test


Case Processing Summary	
	Group	Cases	
		Valid	Missing	Total	
		N	Percent	N	Percent	N	Percent	
Accuracy Rate(AR)-pre Mean	Tai Chi group	28	90.3%	3	9.7%	31	100.0%	
	Control group	27	100.0%	0	0.0%	27	100.0%	
Response Time(RT)-pre Mean	Tai Chi group	28	90.3%	3	9.7%	31	100.0%	
	Control group	27	100.0%	0	0.0%	27	100.0%	
Accuracy Rate(AR)-post Mean	Tai Chi group	28	90.3%	3	9.7%	31	100.0%	
	Control group	27	100.0%	0	0.0%	27	100.0%	
Response Time(RT)-post Mean	Tai Chi group	28	90.3%	3	9.7%	31	100.0%	
	Control group	27	100.0%	0	0.0%	27	100.0%	


Tests of Normality	
	Group	Kolmogorov-Smirnova	Shapiro-Wilk	
		Statistic	df	Sig.	Statistic	df	Sig.	
Accuracy Rate(AR)-pre Mean	Tai Chi group	.087	28	.200*	.979	28	.831	
	Control group	.145	27	.150	.967	27	.518	
Response Time(RT)-pre Mean	Tai Chi group	.145	28	.135	.941	28	.119	
	Control group	.160	27	.074	.961	27	.392	
Accuracy Rate(AR)-post Mean	Tai Chi group	.124	28	.200*	.948	28	.179	
	Control group	.131	27	.200*	.966	27	.490	
Response Time(RT)-post Mean	Tai Chi group	.111	28	.200*	.970	28	.575	
	Control group	.141	27	.180	.961	27	.387	

*. This is a lower bound of the true significance.	
a. Lilliefors Significance Correction	


Accuracy Rate(AR)-pre Mean

Histograms


Normal Q-Q Plots


Response Time(RT)-pre Mean

Histograms


Normal Q-Q Plots


Accuracy Rate(AR)-post Mean

Histograms


Normal Q-Q Plots


Response Time(RT)-post Mean

Histograms


Normal Q-Q Plots
